# MEK inhibition achieves robust and durable responses in Erdheim–Chester disease

**DOI:** 10.1002/hem3.70499

**Published:** 2026-10-06

**Authors:** Francesco Pegoraro, Félicien Triboulet, Matthias Papo, Francesco Peyronel, Anita Argentieri, Jerome Razanamahery, Ahmed Idbaih, Polyzois Makras, Ofer Shpilberg, Oshrat Hershkovitz‐Rokah, Michael Girschikofsky, Matthew Collin, Kristian Bowles, Satyen H. Gohil, Rodothea Amerikanou, Emmanuel Ledoult, Tanguy Le Scornet, Mathilde de Menthon, Etienne Riviere, Xavier Boulu, Henry Dupuy, Achille Aouba, Stanislas Faguer, Xavier Solanich, Elena Sieni, Stéphane Barete, Chiara Bellino, Alessandro Tomelleri, Corrado Campochiaro, Lorenzo Dagna, Zahir Amoura, Jean‐François Emile, Fleur Cohen‐Aubart, Augusto Vaglio, Julien Haroche

**Affiliations:** ^1^ Department of Experimental and Clinical Medicine University of Florence Florence Italy; ^2^ Department of Hematology and Oncology Meyer Children's Hospital IRCCS Florence Italy; ^3^ Internal Medicine Department 2 Institut E3M, French Reference Centre for Histiocytosis, Pitié‐Salpȇtrière Hospital, CIMI INSERM‐UMRS 1135, Sorbonne University, Assistance Publique‐Hôpitaux de Paris Paris France; ^4^ Clinical Pediatrics, Department of Molecular Medicine and Development University of Siena Siena Italy; ^5^ Department of Internal Medicine and Clinical Immunology Dijon University Hospital Dijon France; ^6^ Neuro‐Oncology Department Sorbonne University, Paris Brain Institute ‐ ICM, Inserm, CNRS, Pitié‐Salpêtrière Hospital, DMU Neurosciences, Assistance Publique‐Hôpitaux de Paris Paris France; ^7^ Department of Endocrinology and Diabetes and LCH and ECD Adult Clinic 251 Hellenic Air Force & VA General Hospital Athens Greece; ^8^ Adelson School of Medicine Ariel University Ariel Israel; ^9^ Institute of Hematology, Assuta Medical Centers Tel‐Aviv Israel; ^10^ Department of Molecular Biology Faculty of Natural Sciences Ariel University Ariel Israel; ^11^ Translational Research Lab Assuta Medical Centers Tel‐Aviv Israel; ^12^ Division of Hematology With Stem Cell Transplantation, Hemostaseology and Medical Oncology, Department of Internal Medicine I Ordensklinikum Linz Elisabethinen Linz Austria; ^13^ Faculty of Medical Sciences Newcastle University Newcastle Upon Tyne United Kingdom; ^14^ Norwich Medical School University of East Anglia Norwich United Kingdom; ^15^ Deparment of Hematology University College London Hospitals NHS Foundation Trust London United Kingdom; ^16^ Department of Internal Medicine Lille University Hospital Lille France; ^17^ Department of Internal Medicine Nantes University Hospital Nantes France; ^18^ Department of Internal Medicine and Clinical Immunology Bicetre Hospital, Assistance Publique ‐ Hôpitaux de Paris Le Kremlin Bicêtre France; ^19^ Department of Internal Medicine Haut‐Lévèque University Hospital Bordeaux France; ^20^ Department of Internal Medicine Amiens University Medical Center Amiens France; ^21^ Department of Internal Medicine Ramsay Santé Clinique Aguiléra Biarritz France; ^22^ Department of Internal Medicine Caen University Hospital Caen France; ^23^ Department of Nephrology and Organ Transplantation National Referral Centre for Rare Kidney Diseases‐SORARE, Toulouse University Hospital Toulouse France; ^24^ Department of Internal Medicine Bellvige University Hospital, Hospitalet de Llobregat Barcelona Spain; ^25^ Unit of Dermatology Pitié‐Salpȇtrière Hospital, Assistance Publique‐Hôpitaux de Paris Sorbonne University Paris France; ^26^ Unit of Immunology, Rheumatology, Allegy and Rare Diseases San Raffaele Hospital IRCCS Vita‐Salute San Raffaele University Milano Italy; ^27^ EA4340 BECCOH, Versailles SQY University, Pathology Department, Ambroise Paré Hospital Assistance Publique‐Hôpitaux de Paris Boulogne France; ^28^ Department of Biomedical, Experimental and Clinical Sciences “Mario Serio” University of Florence Florence Italy; ^29^ Nephrology Unit Meyer Children's Hospital IRCCS Florence Italy

## Abstract

Erdheim–Chester disease (ECD) is a rare histiocytic neoplasm characterized by heterogeneous clinical manifestations and limited evidence to guide targeted therapy. While MEK inhibitors (MEKi) are increasingly used, data on their real‐world efficacy, durability, and safety remain incomplete. We analyzed outcomes of patients with ECD treated with MEKi monotherapy across eight countries and assessed the dynamics and predictors of response, treatment retention, and the safety profile of MEKi. We included 170 patients and were able to evaluate response in 159 (94%) of them. Objective responses were observed in 112 patients (70%), disease stabilization in 35 (22%), and disease progression in 12 (8%). Response rates were independent of treatment lines and underlying molecular alterations. The median time to best response was 14 months, with an estimated 2‐year probability of response of 61% (95% CI 51%–67%). In multivariable analysis, dyslipidemia (odds ratio [OR] 0.359, 95% CI 0.171–0.745) and neurodegeneration (OR 0.354, 95% CI 0.144–0.864) were associated with a lower probability of response to MEKi, while cutaneous and gastrointestinal involvement with a higher probability. After a median follow‐up of 27 months (interquartile range [IQR] 12–57), the treatment retention rate was 76%, with most discontinuations attributable to toxicity. Adverse events were reported in 106 patients (62%) and were severe in 39 patients (23%). The estimated 2‐year event‐free survival was 65% (95% CI 57%–72%), and the estimated 2‐year overall survival was 80% (95% CI 72%–86%). MEKi monotherapy provides robust and durable clinical efficacy in patients with ECD, with high response rates and favorable long‐term outcomes, despite a substantial burden of toxicity.

## Graphical abstract

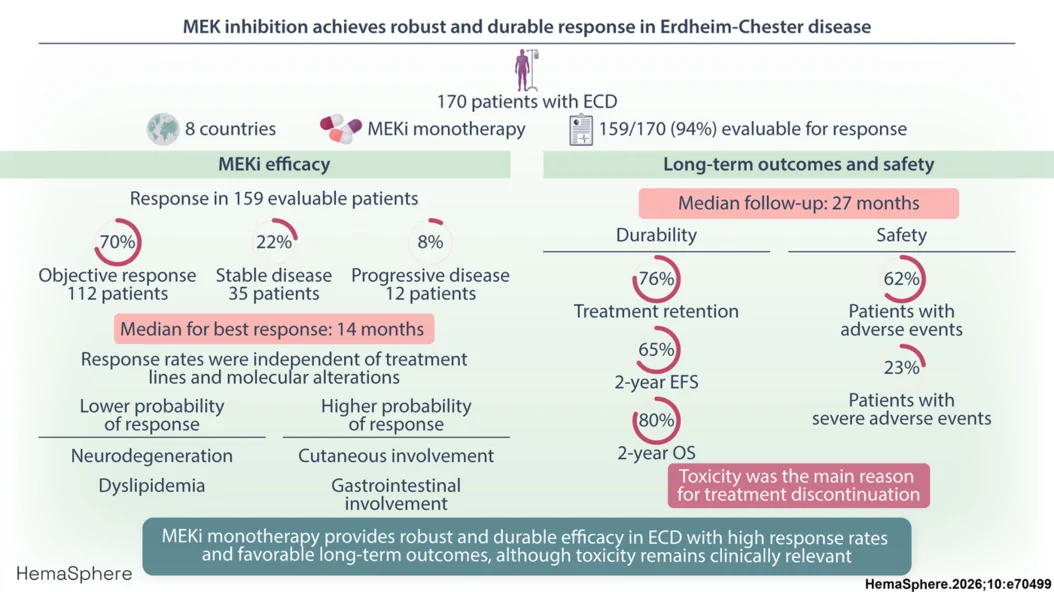

## INTRODUCTION

Erdheim–Chester disease (ECD) is a rare histiocytic cell neoplasm of the L group driven by activating somatic mutations in genes of the MAPK pathway.^1^, ^2^ Its clinical presentation is heterogeneous, frequently including life‐threatening features that require effective therapeutic strategies.^3^, ^4^ In the last decade, treatments targeting mutations in genes of the MAPK pathway (MAPK inhibitors, MAPKi), such as BRAF inhibitors (BRAFi) and MEK inhibitors (MEKi), have revolutionized the management and prognosis of patients with ECD.^5^, ^6^, ^7^ Compared to conventional treatments such as chemotherapy and pegylated interferon‐alpha, MAPKi demonstrated high rates of metabolic responses and rapidly became first‐choice therapeutic options, at least in certain high‐income countries.^3^ However, the long‐term efficacy and tolerability of MAPKi are less established.

We recently demonstrated the sustained efficacy and acceptable safety profile of BRAFi in a large cohort of 172 patients with ECD followed in France and Italy. Interestingly, multisystem ECD (i.e., involving four or more sites) was associated with a higher probability of response to BRAFi.^8^ Similar responses to BRAFi monotherapy were also observed in a US cohort of 64 patients.^9^ We also confirmed, in a cohort of 80 patients with ECD followed at the same institutions, responses to MEKi monotherapy regardless of the underlying mutation.^10^ However, we were not able to assess the dynamics of response, the long‐term toxicity profile, and the potential predictors of MEKi efficacy.

In this study, through an international collaboration within ECD care centers across Europe and Israel, we were able to enroll a substantial cohort of ECD patients treated with MEKi monotherapy. We here report the long‐term efficacy and tolerability and sought to identify baseline features associated with response to MEKi, in order to improve therapeutic strategies in newly diagnosed patients with ECD.

## METHODS

### Patients and outcomes

We screened patients with biopsy‐proven ECD or mixed histiocytosis (ECD‐Langerhans cell histiocytosis [LCH] or ECD‐Rosai–Dorfman–Destombes disease [RDD]) from eight countries. Patients were diagnosed according to international consensus guidelines for ECD.^3^ We included patients who received MEKi monotherapy as any line of therapy and had available data on treatment and outcome, and a minimum follow‐up of 6 months. Data were collected by local physicians and included demographics, disease phenotypes, somatic mutations, treatment regimens and response dynamics, toxicity, retention rates, and survival. Organ involvement was defined according to the latest consensus recommendations for ECD.^3^ Outcomes included response to MEKi monotherapy, time to best response, retention rates, toxicities, and event‐free and overall survival. None of the patients was enrolled in a clinical trial. The study was conducted in accordance with the Declaration of Helsinki and its later amendments.^11^

### Response and safety assessment

The response to treatment was assessed through medical chart review, including clinical and radiologic/metabolic data. Response was assessed in patients receiving at least 3 months of treatment and was defined as a composite endpoint, which included clinical, radiologic,^12^ and metabolic^13^ response criteria, as previously reported.^14^, ^15^, ^16^ Complete response (CR) was defined as a complete clinical, radiologic (magnetic resonance imaging [MRI] or computed tomography [CT]), and metabolic (positron emission tomography, PET) regression of lesions. Partial response (PR) was defined as (a) the resolution of symptoms with stable radiology or metabolic studies; (b) a diameter reduction of at least 30% of the target lesions on radiology studies without appearance of new lesions; or (c) complete or partial metabolic response (resolution or decline ≥30% of 18F‐fluorodeoxyglucose uptake, respectively) with persistent clinical or radiology involvement. Progressive disease (PD) was defined as the development of new ECD‐related manifestations, as a lesion diameter increase of at least 20% or appearance of new lesions on radiologic studies, or as an increase of at least 30% in target lesion activity or appearance of new lesions on PET scans. Stable disease (SD) was defined as neither CR, PR, nor PD. Adverse events (AEs) during MEKi monotherapy were evaluated according to the CTCAE criteria.^17^

### Statistical analysis

Continuous variables were reported as median and interquartile range (IQR), whereas categorical variables as numbers or fractions and percentages. Survival was analyzed using Kaplan–Meier curves. Event‐free survival (EFS) was defined as the time from treatment initiation to the first occurrence of disease progression, MEKi discontinuation, or death from any cause, with patients censored at last follow‐up. Progression‐free survival (PFS) was defined as the time from treatment initiation to disease progression or death from any cause, whichever occurred first.

Time‐dependent response was defined as a time‐to‐response endpoint assessed using a hazard ratio (HR)–based analysis. Univariate Cox proportional odds and hazard analyses were performed for all candidate covariates, followed by the development of a multivariate Cox model through a data‐driven forward selection procedure using the Bayesian information criterion as the stopping rule. Analyses were performed using R software (version 4.5.1) within the RStudio environment (Posit Software, Boston, MA, USA), and plots were created using STATA (version 19). All statistical tests were two‐tailed, and a P‐value < 0.05 was considered statistically significant.

## RESULTS

We screened 206 patients with ECD who received MEKi monotherapy in eight countries (France, Italy, United Kingdom, Israel, Austria, Germany, Greece, and Spain). Twenty‐four patients (12%) were excluded because they were enrolled in a clinical trial, seven (3%) because they did not reach the first response assessment, and five (2%) because of a lack of follow‐up data. Ultimately, 170 patients were included in the study (Figure S1).

### Patients' characteristics

The clinical characteristics of the 170 included patients are detailed in Table 1. Most patients were males (71%), and the median age at ECD diagnosis was 63 years (IQR 51–69). Cardiovascular risk factors included smoking (former or current) in 58 patients (34%), Type 2 diabetes mellitus in 42 (25%), obesity in 27 (16%), dyslipidemia in 54 (32%), hypertension in 84 (49%), and coronary artery disease in 43 (25%). Multisystem ECD (i.e., >4 involved sites) was found in 118 patients (69%), with a median number of involved sites of four (IQR 3–6). Most frequently, ECD involved the bone (*n* = 125, 74%), the perirenal area (*n* = 113, 66%), the large vessels (*n* = 94, 55%), the heart (*n* = 67, 39%), and the central nervous system (CNS; *n* = 64, 38%). In particular, 43 patients (25%) had right atrium infiltration, and 25 (15%) neurodegeneration. Involvement of the lungs (*n* = 61, 36%), skin (*n* = 53, 31%), facial/orbit area (*n* = 50, 29%), hypothalamic/pituitary axis (*n* = 46, 27%), lymph nodes (*n* = 29, 17%), and digestive tract (*n* = 25, 15%) was also reported. Most patients had elevated CRP at diagnosis (*n* = 112/138, 81%) and clonal hematopoiesis (*n* = 66/115, 57%). Associated diseases included mixed histiocytosis in 34 patients (20%; ECD‐LCH in 22, ECD‐RDD in 11, and ECD‐LCH‐RDD in 1), and second cancer in 54 (32%; hematologic in 37, 22%; solid in 25, 15%; both solid and hematologic in 8 of them). Somatic mutations were identified in 112 of 164 tested patients (68%) and included *BRAF* ^V600E^ in 57 (35%), *MAP2K1* in 46 (28%), *NRAS* in 6 (3%), and *KRAS* in 4 (2%).

**Table 1:** Clinical characteristics of the 170 patients included in the study.

Age at ECD diagnosis, years	63 (51–69)
Female sex	49 (29)
Cardiovascular comorbidities	
Smoking (current or former)	58 (34)
T2DM	42 (25)
Obesity	27 (16)
Dyslipidemia	54 (32)
Hypertension	84 (49)
CAD	43 (25)
Organ involvement	
Long bone	125 (74)
CNS	64 (38)
Neurodegeneration	25 (15)
Facial/orbit	50 (29)
Heart	67 (39)
Right atrium	43 (25)
Large vessel	94 (55)
Lung	61 (36)
Perirenal	113 (66)
Hypothalamic/pituitary	46 (27)
Skin	53 (31)
Gastrointestinal	25 (15)
Lymph nodes	29 (17)
Number of involved sites	4 (3–6)
≥4 sites involved	118 (69)
Elevated CRP	112/138 (81)
Clonal hematopoiesis	66/115 (57)
Mixed histiocytosis	34 (20)
ECD‐LCH	23 (14)
ECD‐RDD	12 (7)
Second cancer	54 (32)
Hematologic	37 (22)
Solid	25 (15)
Somatic mutations^a^	
*BRAF* ^V600E^	57 (35)
*MAP2K1*	46 (28)
*NRAS*	7 (4)
*KRAS*	6 (3)
No mutation	51 (30)

*Note*: Data are *n* (%) or median (interquartile range [IQR]).

Abbreviations: CAD, coronary artery disease; CNS, central nervous system; CRP, C‐reactive protein; ECD, Erdheim–Chester disease; LCH, Langerhans cell histiocytosis; RDD, Rosai–Dorfman–Destombes disease; T2DM, Type 2 diabetes mellitus.

^a^ Out of 164 tested patients.

### Treatment and response

Most patients received cobimetinib (*n* = 136, 80%) or trametinib (*n* = 31, 18%), while only three patients (2%) received binimetinib due to intolerance to the other two molecules. These drugs were started on a standard dose for histiocytosis, namely 20 mg bid (3 weeks on, 1 week off) for cobimetinib and 2 mg daily for trametinib. MEKi were used as first line in 83 patients (49%), second line in 57 (34%), and third line in 30 (17%) (Figure S1). Previous lines of treatment mainly included interferon‐alpha (*n* = 53), BRAFi (*n* = 32), IL1 receptor antagonists (*n* = 12), and chemotherapy (*n* = 10).

The median time from diagnosis to MEKi start was 8 months (IQR 2–31). We could not assess response to MEKi in 11 patients (Table S1), due to early (<3 months) discontinuation (*n* = 10), or because they deceased soon after treatment start of ECD‐ and treatment‐unrelated causes (*n* = 1). Of note, none of these 11 patients progressed while on MEKi. Reasons for early discontinuation included gastrointestinal symptoms (*n* = 4), skin rash (*n* = 2), cardiac toxicity (*n* = 1), hypertension (*n* = 1), urinary tract infections (*n* = 1), and dropped head syndrome (*n* = 1). No statistical differences were found in baseline features between patients who discontinued early and those who were assessable for response (Table S1).

Of the 159 patients (94%) in whom we were able to assess the response to MEKi, objective responses were observed in 112 (70%; CR in 46 [29%] and PR in 66 [42%]), while 35 (22%) remained stable, and 12 (8%) progressed. Responses were observed irrespective of the line of treatment (P = 0.122) and the presence of underlying mutations in genes of the MAPK pathway (P = 0.703; Figure 1A). The estimated 2‐year probability of response was 61% (95% CI 51%–67%), and the median time to best response was 14 months (Figure 1B). The clinical characteristics of the 12 patients who progressed on MEKi are detailed in Table S2.

**Figure 1:**
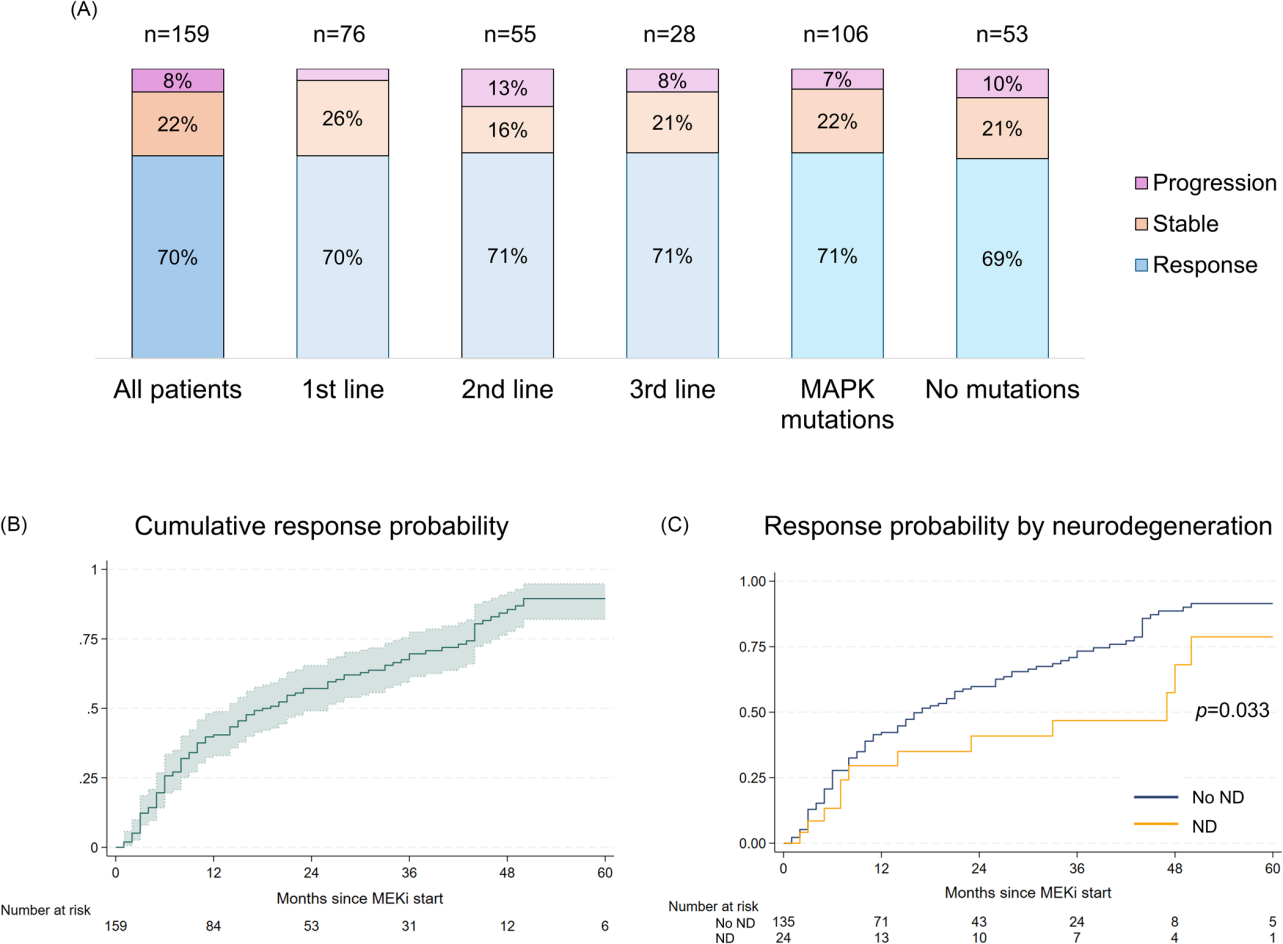
**Response to MEK inhibitors (MEKi).** Response to MEKi monotherapy in the 159 Erdheim–Chester disease (ECD) patients with available data **(A)**; cumulative response probability in the whole cohort **(B)**, and in patients with and without neurodegeneration **(C)**.

### Predictors of response

We evaluated baseline features that were potentially associated with the probability of response to MEKi monotherapy (Table 2). In the unadjusted analysis, we found that dyslipidemia (odds ratio [OR] 0.359, 95% CI 0.171–0.745; P = 0.006) and neurodegeneration (OR 0.354, 95% CI 0.144–0.864; P = 0.022) were associated with a lower response probability. Consistently, the estimated 2‐year probability of response was significantly lower in patients with neurodegeneration (log rank P = 0.033; Figure 1C). Conversely, cutaneous involvement was associated with a higher probability of response to MEKi (OR 2.346, 95% CI 1.065–5.613; P = 0.042). In the multivariable analysis, dyslipidemia (OR 0.267, 95% CI 0.115–0.596; P = 0.002) and neurodegeneration (OR 0.307, 95% CI 0.108–0.853; P = 0.024) were confirmed as predictors of non‐response to MEKi monotherapy. Moreover, in addition to cutaneous involvement (OR 3.749, 95% CI 1.463–10.95; P = 0.009), gastrointestinal disease was associated with a higher probability of response (OR 6.512, 95% CI 1.646–44.10; P = 0.019; Table 2).

**Table 2:** Predictors of response to MEK inhibitors (MEKi) in the 159 evaluable patients.

	r1/N1 (%)	r2/N2 (%)	Univariable		Multivariable	
			OR (95% CI)	P‐value	OR (95% CI)	P‐value
Age at diagnosis	‐	‐	0.976 (0.949–1.001)	0.073		
Female sex	33/46 (72)	79/113 (70)	1.093 (0.520–2.385)	0.819		
CV comorbidities					0.267 (0.115–0.596)	0.002
Smoking	33/50 (66)	65/90 (72)	0.834 (0.560–1.251)	0.373		
T2DM	24/37 (65)	82/114 (72)	0.720 (0.330–1.616)	0.415		
Obesity	16/26 (62)	90/124 (73)	0.604 (0.252–1.500)	0.264		
Dyslipidemia	28/50 (56)	78/100 (78)	0.359 (0.171–0.745)	0.006		
Hypertension	51/77 (66)	55/73 (75)	0.642 (0.312–1.301)	0.222		
CAD	24/40 (60)	82/111 (74)	0.530 (0.284–1.146)	0.103		
Organ involvement						
Long bone	81/117 (69)	30/41 (73	0.825 (0.361–1.790)	0.635		
CNS	40/62 (65)	71/96 (74	0.640 (0.320–1.282)	0.206		
Neurodegeneration	12/24 (50)	99/134 (74	0.354 (0.144–0.864)	0.022	0.307 (0.108–0.853)	0.024
Facial/orbit	35/47 (75)	76/111 (68	1.343 (0.634–2.980)	0.452		
Heart	44/63 (70)	68/96 (71	0.954 (0.477–1.928)	0.893		
Right atrium	29/40 (73)	83/119 (70	1.143 (0.526‐2.618)	0.742		
Large vessel	67/88 (76)	45/71 (63	1.843 (0.929–3.698)	0.082		
Lung	41/61 (67)	71/98 (72	0.639 (0.317–1.296)	0.211		
Perirenal	72/107 (67)	40/52 (77	0.617 (0.280–1.296)	0.214		
Hypothalamic/pituitary	31/43 (72)	80/115 (70	1.130 (0.529–2.523)	0.757		
Skin	40/49 (82)	72/110 (65)	2.346 (1.065–5.613)	0.042	3.749 (1.463–10.95)	0.009
Gastrointestinal	20/23 (87)	92/136 (68)	3.188 (1.024–14.40)	0.073	6.512 (1.646–44.10)	0.019
Lymph nodes	21/27 (78)	91/132 (69)	1.577 (0.623–4.577)	0.362		
Number of involved sites	‐	‐	1.125 (0.928–1.380)	0.24		
≥4 involved sites	81/111 (73)	31/48 (65)	1.481 (0.710–3.045)	0.289		
Elevated CRP	74/101 (73)	18/26 (69)	1.218 (0.455–3.057)	0.682		
Clonal hematopoiesis	41/61 (67)	34/47 (72)	0.784 (0.335–1.791)	0.567		
Mixed histiocytosis						
ECD‐LCH	14/22 (64)	98/137 (72)	0.696 (0.275–1.864)	0.453		
ECD‐RDD	8/12 (67)	103/146 (71)	0.835 (0.249–3.262)	0.778		
Second cancer						
Hematologic	23/35 (66)	89/124 (72)	0.754 (0.343–1.716)	0.489		
Solid	19/24 (79)	93/134 (69)	1.675 (0.624–5.326)	0.336		
Somatic mutations						
*BRAF* ^V600E^	34/53 (64)	74/100 (74	0.629 (0.307–1.296)	0.205		
*MAP2K1*	33/43 (77)	75/107 (70	1.408 (0.635–3.319)	0.413		
Other	10/12 (83)	102/146 (70	2.157 (0.540–14.41)	0.334		
Any mutation	75/105 (71)	37/54 (69)	1.149 (0.556–2.331)	0.703		
Treatment line			1.059 (0.548–2.144)	0.867		
First	53/76 (70)	‐				
Second	39/55 (71)	‐				
Third	20/28 (71)	‐				

Abbreviations: CAD, coronary artery disease; CNS, central nervous system; CRP, C‐reactive protein; CV, cardiovascular; ECD, Erdheim–Chester disease; LCH, Langerhans cell histiocytosis; OR, odds ratio; RDD, Rosai–Dorfman–Destombes disease; T2DM, Type 2 diabetes mellitus.

We also assessed factors associated with earlier responses (Table S3). At univariable analysis, right atrium infiltration (HR 1.643, 95% CI 1.073–2.517; P = 0.022), gastrointestinal involvement (HR 1.739, 95% CI 1.071–2.826; P = 0.025) were associated with earlier response, while CNS infiltration (HR 0.605, 0.409–0.897; P = 0.012) and neurodegeneration (HR 0.524, 0.286–0.957; P = 0.036) with less rapid responses. At multivariable analysis, right atrium infiltration (HR 3.959, 95% CI 1.956–8.014; P = 0.001) and gastrointestinal involvement (HR 2.466, 95% CI 1.279–4.753; P = 0.007) were confirmed as predictors of earlier response, while hypertension (HR 0.441, 95% CI 0.238–0.819; P = 0.009) and the latency of treatment (i.e., third‐line vs. second‐, vs. first; HR 0.545, 95% CI 0.358–0.830; P = 0.005) were also associated with an inferior probability of earlier response (Table S3).

### Outcomes

At the last follow‐up, 130 patients (76%) were still receiving MEKi monotherapy. The remaining 40 patients permanently discontinued treatment after a median duration of 5 months (IQR 2.5–12). Discontinuations were mostly due to toxicity (*n* = 25, 63%); in the remaining cases, they occurred due to second cancer (*n* = 7), progression (*n* = 4), decision of the patient (*n* = 2), severe infections (*n* = 1), and switch to BRAFi after identification of a *BRAF* ^V600E^ mutation (*n* = 1).

After a median follow‐up of 27 months (IQR 12–57) since MEKi initiation, 43 patients (25%) deceased. Causes of death were ECD‐related in 21 of them (48%), including severe infections in seven, multisystem ECD progression in five, and cardiac (*n* = 4), peri‐renal (*n* = 3), and CNS (*n* = 2) disease in the remaining nine. The estimated 2‐ and 5‐year probability of survival were 80% (95% CI 72%–86%) and 70% (95% CI 60%–78%), respectively (Figure 2A). Events were observed in 71 patients (42%) and included deaths in 23 (32%), progression in 12 (17%), and withdrawal in 36 (51%). The estimated 2‐year EFS probability was 65% (95% CI 57%–72%; Figure 2B), while the estimated 2‐year PFS probability was 77% (95% CI 69%–84%; Figure 2C).

**Figure 2:**
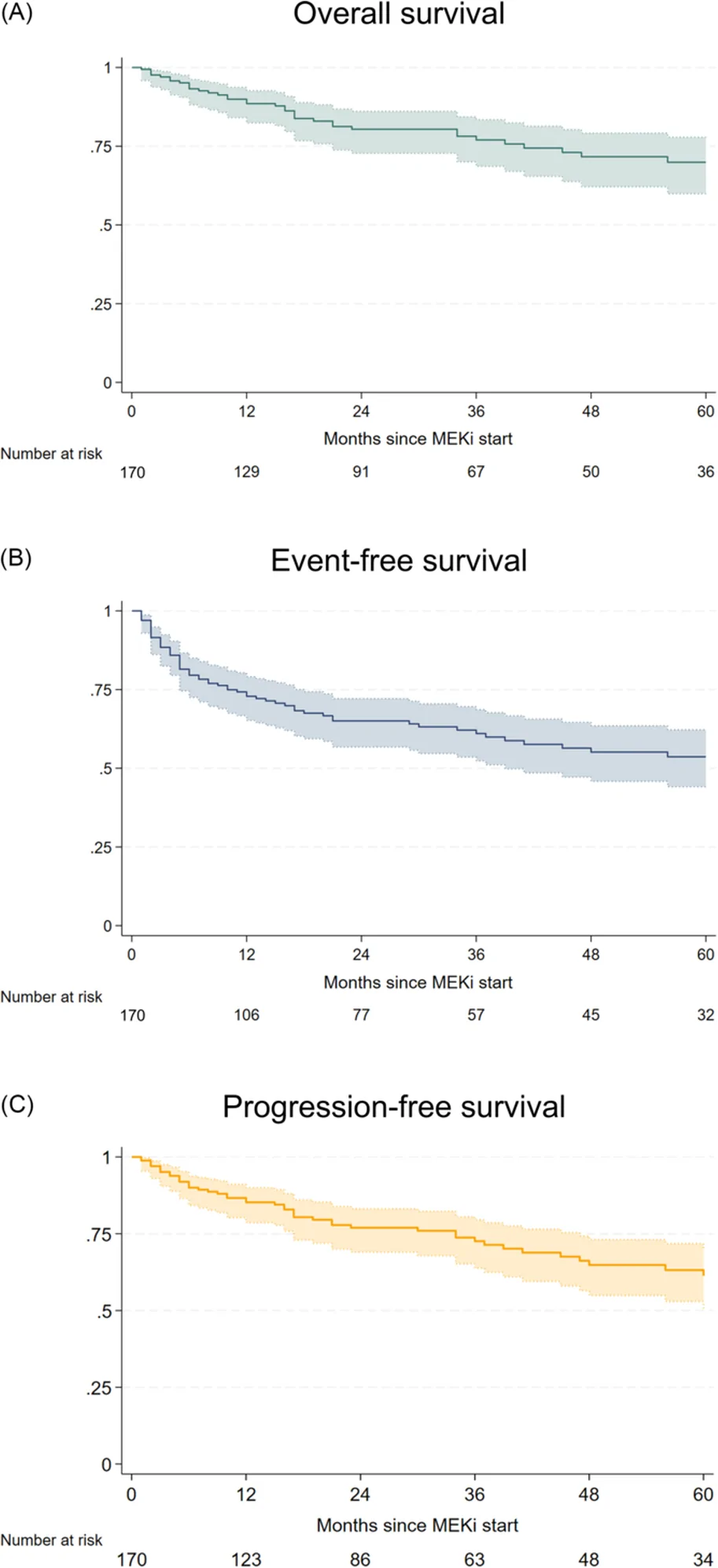
**Survival estimated 5‐year overall (A), event‐free (B), and progression‐free (C) survival in the 170 included patients.** MEKi, MEK inhibitors.

### Treatment‐related toxicity

AEs were reported in 106 patients (62%), including Grade 1–2 events in 67 (39%) and Grade 3–4 in 39 (23%). In 32 patients (19%), AEs required permanent dose reduction, while in 25 (15%), permanent discontinuation (Table 3). In 18 additional patients (11%), switching to another MEKi (cobimetinib to trametinib in 15, and trametinib to binimetinib in three) was necessary to mitigate toxicity and guarantee drug retention. The most frequently observed AEs were cutaneous (*n* = 49, 29% of the cohort) and mostly consisted of acneiform rash. Musculoskeletal (*n* = 22, 13%, mostly due to creatine kinase elevation but also two cases of dropped head syndrome), gastrointestinal (*n* = 20, 12%; mostly consisting of diarrhea and abdominal pain), and systemic (*n* = 14, 8%) events were also frequently reported. Severe AEs (grade 3‐4) were more frequently cutaneous (*n* = 8; no cancer), systemic (*n* = 8), gastrointestinal (*n* = 7), musculoskeletal (*n* = 6), and cardiac (*n* = 5; Table 3). However, none of the deaths were attributed to treatment toxicity.

**Table 3:** Adverse events.

	Total	Grades 1–2^a^	Grades 3–4^a^
At least one adverse event	106 (62)	67 (39)	39 (23)
Adverse events requiring treatment withdrawal	25 (15)	10 (6)	15 (9)
Adverse events requiring dose reduction	32 (19)	27 (16)	5 (3)
Skin	49 (29)	41 (24)	8 (5)
Acneiform rash	46 (27)	39 (23)	7 (4)
Photosensitivity	3 (2)	3 (2)	0 (0)
Maculopapular lesions	3 (2)	2 (1)	1 (1)
Dry skin	1 (1)	1 (1)	0 (0)
Musculoskeletal	23 (14)	16 (9)	7 (4)
Creatine kinase elevation	19 (11)	14 (8)	5 (3)
Musculoskeletal pain	4 (2)	4 (2)	0 (0)
Myositis	3 (2)	0 (0)	3 (2)
Hepatic	7 (4)	4 (2)	3 (2)
Liver cytolysis	6 (4)	4 (2)	2 (1)
Cholestasis	1 (1)	0 (0)	1 (1)
Gastrointestinal	20 (12)	14 (8)	7 (4)
Diarrhea	15 (9)	11 (6)	4 (2)
Abdominal pain	5 (3)	4 (2)	1 (1)
Vomiting	2 (1)	1 (1)	1 (1)
Hematemesis	1 (1)	0 (0)	1 (1)
Colitis	1 (1)	0 (0)	1 (1)
Cardiac	6 (4)	1 (1)	5 (3)
Cardiac function impairment	6 (4)	1 (1)	5 (3)
Infections	3 (2)	3 (2)	0 (0)
Urinary tract	1 (1)	1 (1)	0 (0)
Zoster	1 (1)	1 (1)	0 (0)
Pneumonia	1 (1)	1 (1)	0 (0)
Ocular	5 (3)	2 (1)	3 (2)
Retinitis	5 (3)	2 (1)	3 (2)
Systemic	14 (8)	6 (4)	8 (5)
Edema	7 (4)	4 (2)	3 (2)
Fatigue	2 (1)	1 (1)	1 (1)
Fever	2 (1)	1 (1)	1 (1)
Hypertension	3 (2)	0 (0)	3 (2)
Pulmonal embolism	1 (1)	1 (1)	0 (0)
Hematologic	3 (2)	2 (1)	1 (1)
Pancytopenia	1 (1)	0 (0)	1 (1)
Thrombocytopenia	1 (1)	1 (1)	0 (0)
Eosinophilia	1 (1)	1 (1)	0 (0)
Renal	2 (1)	0 (0)	2 (1)
Acute kidney failure	1 (1)	0 (0)	1 (1)
Nephrotic syndrome	1 (1)	0 (0)	1 (1)
Neurologic	2 (1)	2 (1)	0 (0)
Seizure	1 (1)	1 (1)	0 (0)
Vertigo	1 (1)	1 (1)	0 (0)

^a^ According to the CTCAE criteria.

## DISCUSSION

In this real‐world study, we demonstrated the long‐term efficacy and tolerability of MEKi monotherapy in a large cohort of patients with ECD. We also characterized response dynamics and identified predictors of both overall and time‐dependent response.

ECD is a difficult‐to‐treat disease with a dismal prognosis in historical cohorts.^18^, ^19^ The introduction of the BRAFi vemurafenib, following the discovery of *BRAF* ^V600E^ mutations in ECD, has substantially improved outcomes in patients harboring this mutation. However, until the first description of cobimetinib efficacy in ECD and other histiocytic disorders,^7^ patients with *BRAF* ^WT^ disease lacked comparably effective options. Diamond et al. showed that cobimetinib induced rapid metabolic responses in 18 patients with histiocytosis (including 12 ECD), irrespective of the underlying mutations.^7^ A similar response profile was shown in 28 patients with non‐Langerhans cell histiocytosis (including 17 ECD) treated with trametinib, another MEKi.^20^ These molecules were recognized as first‐line options for patients with *BRAF* ^WT^ disease and aggressive clinical presentations,^3^ and are increasingly used worldwide. Nevertheless, considerable heterogeneity remains regarding indications and dosing of MEKi in clinical practice, and long‐term efficacy and safety data are limited.

In this study, we analyzed responses to MEKi in a large cohort of 159 ECD patients. Objective responses were observed in 70% of patients, regardless of treatment line or underlying molecular alterations, consistent with previous reports.^8^, ^21^ Most of the remaining patients achieved disease stabilization, with an estimated 2‐year PFS probability of 94%. Responses were generally rapid, with a median time to best response of 14 months and were sustained over time.

We also identified predictors of non‐response to MEKi, notably dyslipidemia and neurodegeneration. The latter is a potentially severe manifestation of ECD, occurring in approximately 15%–20% of patients.^4^, ^22^ It is typically characterized by a progressive course, and responses to treatments, including MEKi, are often inferior compared with other disease manifestations. This may reflect the largely irreversible nature of neurodegenerative lesions as well as limited or variable CNS penetration of targeted therapies, as demonstrated in LCH.^23^ Combination therapy with BRAFi and MEKi may improve disease control in selected patients with CNS involvement, potentially by enhancing pathway suppression and mitigating resistance. In addition, alternative targeted approaches such as CSF1R inhibitors have shown preclinical activity in neurodegenerative ECD and may represent a therapeutic option for patients who are refractory to MAPKi.^24^

On the other hand, the association between dyslipidemia and reduced response is most plausibly explained by the concomitant use of lipid‐lowering agents, particularly statins. Although preclinical studies have suggested synergistic pro‐apoptotic effects of MEKi and statins,^25^ real‐world studies in metastatic melanoma have identified clinically relevant drug–drug interactions between these agents, which were associated with inferior outcomes.^26^ In our study, we had no data on concomitant statin use. Therefore, we cannot ascertain if the observed association between dyslipidemia and poor response was influenced drug–drug interaction.

Interestingly, we also observed more rapid responses in patients with right atrium infiltration, consistent with previous reports highlighting the efficacy of MAPKi in cardiac ECD.^27^ At present, there is no clear biological explanation for the association between hypertension and time‐dependent response. Nevertheless, the observed association between certain cardiovascular risk factors and poorer treatment response deserves further investigation to determine whether it reflects a true biological phenomenon or residual confounding. Of note, cardiovascular risk factors were included based on previous evidence of their prognostic relevance.^10^, ^28^ Other comorbidities such as chronic kidney disease could not be assessed because these data were unavailable for most patients.

Treatment‐related toxicity remains a major concern in patients with ECD receiving MEKi. In our cohort, the retention rate of MEKi was 76%, with approximately two‐thirds of the discontinuations attributable to toxicity. However, AEs were generally manageable, and no treatment‐related deaths were observed. In some cases, toxicity was mitigated by switching to an alternative MEKi (most commonly from cobimetinib to trametinib or binimetinib). In others, MEKi were combined with anti‐IL1 agents, an approach that has been shown to improve the retention rates of MAPKi in patients with ECD.^29^ Alternatively, another possible strategy to manage treatment‐related toxicities, particularly in patients with durable disease control, could involve treatment holidays, as recently proposed for patients receiving BRAFi.^30^

Conducting randomized clinical trials in rare diseases such as ECD is inherently challenging. Nonetheless, results from the French randomized cobimetinib versus placebo trial (COBRAH, NCT04007848) are awaited and may provide critical data to guide therapeutic indications in Europe. None of the patients included in our study were enrolled in clinical trials, and all received MEKi off‐label, as these agents are not currently approved by the European Medicines Agency (EMA). Collectively, these efforts are likely to contribute meaningfully to future regulatory decisions, representing a major milestone in the management of histiocytoses (in particular ECD), diseases that still lack EMA‐approved therapeutic strategies.

The limitations of this study include its retrospective design, variability in baseline assessments across centers, lack of centralized response adjudication, heterogeneous follow‐up, and the absence of pharmacokinetic data. Nevertheless, the collection of efficacy and safety data for MEKi in such a large cohort of ECD patients is unprecedented.

In conclusion, we demonstrated robust and sustained efficacy of MEKi monotherapy in ECD, with an acceptable toxicity profile. Responses are inferior in patients with neurodegeneration, and potential drug–drug interactions should be carefully considered in clinical practice.

## AUTHOR CONTRIBUTIONS

**Francesco Pegoraro**: Conceptualization; investigation; writing—original draft; methodology; validation; visualization; writing—review and editing; data curation; software; formal analysis. **Félicien Triboulet**: Data curation; investigation; writing—review and editing. **Matthias Papo**: Data curation; investigation; writing—review and editing. **Francesco Peyronel**: Investigation; conceptualization; data curation; formal analysis; writing—original draft; writing—review and editing; methodology; software. **Anita Argentieri**: Data curation; writing—review and editing. **Jerome Razanamahery**: Data curation; writing—review and editing. **Ahmed Idbaih**: Writing—review and editing; data curation. **Polyzois Makras**: Writing—review and editing; data curation. **Ofer Shpilberg**: Writing—review and editing; data curation. **Oshrat Hershkovitz‐Rokah**: Writing—review and editing; data curation. **Michael Girschikofsky**: Data curation; writing—review and editing. **Matthew Collin**: Writing—review and editing; data curation. **Kristian Bowles**: Writing—review and editing; data curation. **Satyen H. Gohil**: Data curation; writing—review and editing. **Rodothea Amerikanou**: Data curation; writing—review and editing. **Emmanuel Ledoult**: Writing—review and editing; data curation. **Tanguy Le Scornet**: Data curation; writing—review and editing. **Mathilde de Menthon**: Writing—review and editing; data curation. **Etienne Riviere**: Data curation; writing—review and editing. **Xavier Boulu**: Data curation; writing—review and editing. **Henry Dupuy**: Data curation; writing—review and editing. **Achille Aouba**: Writing—review and editing; data curation. **Stanislas Faguer**: Data curation; writing—review and editing. **Xavier Solanich**: Data curation; writing—review and editing. **Elena Sieni**: Writing—review and editing; data curation. **Stéphane Barete**: Data curation; writing—review and editing. **Chiara Bellino**: Writing—review and editing; data curation. **Alessandro Tomelleri**: Data curation; writing—review and editing. **Corrado Campochiaro**: Writing—review and editing; data curation. **Lorenzo Dagna**: Data curation; writing—review and editing. **Zahir Amoura**: Writing—review and editing; data curation. **Jean‐François Emile**: Data curation; writing—review and editing; investigation. **Fleur Cohen‐Aubart**: Writing—review and editing; investigation; data curation; supervision. **Augusto Vaglio**: Supervision; conceptualization; investigation; methodology; validation; writing—review and editing; visualization. **Julien Haroche**: Data curation; supervision; validation; methodology; visualization; writing—review and editing; investigation; conceptualization; project administration; resources.

## CONFLICT OF INTEREST STATEMENT

The authors declare no conflicts of interest.

## ETHICS STATEMENT

The study was conducted in accordance with the Declaration of Helsinki and its later amendments. It was approved by the CESREES #2814848 ethical committee (NCT04437381).

## FUNDING

Open access publishing facilitated by Universita degli Studi di Firenze, as part of the Wiley ‐ CRUI‐CARE agreement.

## Supporting information

Supporting Information.

## Data Availability

The data that support the findings of this study are available on request from the corresponding author. The data are not publicly available due to privacy or ethical restrictions.

## References

[1] Pegoraro F , Papo M , Maniscalco V , Charlotte F , Haroche J , Vaglio A . Erdheim–Chester disease: a rapidly evolving disease model. Leukemia. 2020;34(11):2840‐2857. 10.1038/s41375-020-0944-4 32591646

[2] Haroche J , Cohen‐Aubart F , Amoura Z . Erdheim–Chester disease. Blood. 2020;135(16):1311‐1318. 10.1182/blood.2019002766 32107533

[3] Goyal G , Heaney ML , Collin M , et al. Erdheim–Chester disease: consensus recommendations for evaluation, diagnosis, and treatment in the molecular era. Blood. 2020;135(22):1929‐1945. 10.1182/blood.2019003507 32187362

[4] Tesi M , Pegoraro F , Peyronel F , et al. Cluster analysis reveals the clinical spectrum of Erdheim–Chester disease. Leukemia. 2025;39(8):1987‐1996. 10.1038/s41375-025-02656-w PMC12865793 40437172

[5] Haroche J , Cohen‐Aubart F , Emile JF , et al. Reproducible and sustained efficacy of targeted therapy with vemurafenib in patients with BRAF(V600E)‐mutated Erdheim–Chester disease. J Clin Oncol. 2015;33(5):411‐418. 10.1200/JCO.2014.57.1950 25422482

[6] Cohen‐Aubart F , Emile JF , Carrat F , et al. Targeted therapies in 54 patients with Erdheim–Chester disease, including follow‐up after interruption (the LOVE study). Blood. 2017;130(11):1377‐1380. 10.1182/blood-2017-03-771873 28667012

[7] Diamond EL , Durham BH , Ulaner GA , et al. Efficacy of MEK inhibition in patients with histiocytic neoplasms. Nature. 2019;567(7749):521‐524. 10.1038/s41586-019-1012-y PMC6438729 30867592

[8] Pegoraro F , Cohen‐Aubart F , Papo M , et al. MEK inhibitors are effective in Erdheim–Chester disease regardless of MAPK pathway mutations. Leukemia. 2025;39(6):1540‐1542. 10.1038/s41375-025-02614-6 PMC12133560 40269272

[9] Goyal G , Reiner AS , Bossert D , et al. Long‐term outcomes with single‐agent BRAF inhibitor therapy in Erdheim–Chester disease. Blood. 2025;145(18):2100‐2103. 10.1182/blood.2024028032 PMC12105718 40053897

[10] Pegoraro F , Peyronel F , Papo M , et al. Real‐life efficacy and safety of BRAF inhibitors in Erdheim–Chester disease. Am J Hematol. 2026;101(1):182‐186. 10.1002/ajh.70137 41204885

[11] World Medical Association . Declaration of Helsinki: ethical principles for medical research involving human subjects. JAMA. 2013;310(20):2191‐2194. 10.1001/jama.2013.281053 24141714

[12] Eisenhauer EA , Therasse P , Bogaerts J , et al. New response evaluation criteria in solid tumours: revised RECIST guideline (version 1.1). Eur J Cancer. 2009;45:228‐247. 10.1016/j.ejca.2008.10.026 19097774

[13] Wahl RL , Jacene H , Kasamon Y , Lodge MA . From RECIST to PERCIST evolving considerations for PET response criteria in solid tumors. J Nucl Med. 2009;50(S1):122S‐150S. 10.2967/jnumed.108.057307 PMC2755245 19403881

[14] Pegoraro F , Maniscalco V , Peyronel F , et al. Long‐term follow‐up of mTOR inhibition for Erdheim–Chester disease. Blood. 2020;135(22):1994‐1997. 10.1182/blood.2019004478 32299103

[15] Pegoraro F , Mazzariol M , Trambusti I , et al. Childhood‐onset Erdheim–Chester disease in the molecular era: clinical phenotypes and long‐term outcomes of 21 patients. Blood. 2023;142(13):1167‐1171. 10.1182/blood.2023020569 PMC10934279 37490651

[16] Pegoraro F , Papo M , Cohen‐Aubart F , et al. Long‐term outcome and prognosis of mixed histiocytosis (Erdheim–Chester disease and Langerhans cell histiocytosis). EClinicalMedicine. 2024;73:102658. 10.1016/j.eclinm.2024.102658 PMC11152896 38841707

[17] National Cancer Institute . Patient‐Reported Outcomes version of the Common Terminology Criteria for Adverse Events (PRO‐CTCAE). https://healthcaredelivery.cancer.gov/pro‐ctcae

[18] Haroche J , Amoura Z , Dion E , et al. Cardiovascular involvement, an overlooked feature of Erdheim–Chester disease: report of 6 new cases and a literature review. Medicine. 2004;83(6):371‐392. 10.1097/01.md.0000145368.17934.91 15525849

[19] Veyssier‐Belot C , Cacoub P , Caparros‐Lefebvre D , et al. Erdheim–Chester disease. Clinical and radiologic characteristics of 59 cases. Medicine. 1996;75(3):157‐169. 10.1097/00005792-199605000-00005 8965684

[20] Aaroe A , Kurzrock R , Goyal G , et al. Successful treatment of non‐Langerhans cell histiocytosis with the MEK inhibitor trametinib: a multicenter analysis. Blood Adv. 2023;7(15):3984‐3992. 10.1182/bloodadvances.2022009013 PMC10410131 36857436

[21] Acosta‐Medina AA , Abeykoon JP , Zanwar S , et al. Efficacy of MEK inhibitors in Erdheim–Chester disease: impact of MAPK pathway pathogenic variants. Leukemia. 2025;39(4):991‐994. 10.1038/s41375-025-02526-5 PMC11976263 39934281

[22] Cohen Aubart F , Idbaih A , Emile JF , et al. Histiocytosis and the nervous system: from diagnosis to targeted therapies. Neuro‐Oncology. 2021;23(9):1433‐1446. 10.1093/neuonc/noab107 PMC8408883 33993305

[23] Pegoraro F , Chalard F , Evseev D , et al. Efficacy of MAPK inhibitors in children with neurodegenerative Langerhans cell histiocytosis: results from the European Consortium for Histiocytosis. HemaSphere. 2026;10(6):e70392. 10.1002/hem3.70392 PMC13242968 42261376

[24] Vicario R , Fragkogianni S , Pokrovskii M , et al. Role of clonal inflammatory microglia in histiocytosis‐associated neurodegeneration. Neuron. 2025;113(7):1065‐1081.e13. 10.1016/j.neuron.2025.02.007 PMC12459608 40081365

[25] Iizuka‐Ohashi M , Watanabe M , Sukeno M , et al. Blockage of the mevalonate pathway overcomes the apoptotic resistance to MEK inhibitors with suppressing the activation of Akt in cancer cells. Oncotarget. 2018;9(28):19597‐19612. 10.18632/oncotarget.24696 PMC5929411 29731968

[26] Mezi S , Botticelli A , Pomati G , et al. Impact of drug–drug interactions on clinical outcomes in metastatic melanoma patients treated with combined BRAF/MEK inhibitors: a real‐world study. Pigm Cell Melanoma Res. 2025;38(4):e70026. 10.1111/pcmr.70026 PMC12127241 40451782

[27] Azoulay LD , Bravetti M , Cohen‐Aubart F , et al. Prevalence, patterns and outcomes of cardiac involvement in Erdheim–Chester disease. Eur Heart J. 2023;44(26):2376‐2385. 10.1093/eurheartj/ehac741 36545799

[28] Chazal T , Pegoraro F , Manari G , et al. Clinical phenotypes and long‐term outcome of kidney involvement in Erdheim–Chester histiocytosis. Kidney Int. 2023;103(1):177‐186. 10.1016/j.kint.2022.09.027 36374823

[29] Campochiaro C , Tomelleri A , Catamerò F , Pegoraro F , Vaglio A , Dagna L . Anakinra improves retention rate of targeted treatments in Erdheim–Chester disease. Rheumatology. 2025;64(8):4722‐4725. 10.1093/rheumatology/keaf023 39814074

[30] Pegoraro F , Catamerò F , Allegretta F , et al. Treatment holidays in patients with Erdheim–Chester disease receiving vemurafenib: a prospective pilot study. Blood Adv. 2025;9(9):2119‐2121. 10.1182/bloodadvances.2024015399 PMC12051115 39787607

