# Supplementary material for: MEK inhibition achieves robust and durable responses in Erdheim–Chester disease
Source: Hemasphere. 2026 Oct 6;10(10):e70499. doi: 10.1002/hem3.70499 (PMC13641181; doi:10.1002/hem3.70499)
Supplement: Supplementary file 1 [file HEM3-10-e70499-s001.docx]

**Figure S1**. Flow diagram of patients’ inclusion and outcomes

*Abbreviations: MEKi, MEK inhibitors*

**Table S1.** Clinical characteristics of patients evaluable and not evaluable for response to MEK inhibitors

|  | **Evaluable for response to MEKi (n=159)** | **Not evaluable for response to MEKi (n=11)** |
| --- | --- | --- |
| Age at ECD diagnosis, years | 63 (51-69) | 65 (45-73) |
| Female sex | 46 (29) | 3 (27) |
| Organ involvement  Long bone  CNS  Neurodegeneration  Facial/orbit  Heart  Right atrium  Large vessel  Lung  Perirenal  Hypothalamic/Pituitary  Skin  Gastrointestinal  Lymph nodes  Number of involved sites  ≥4 sites involved | 117 (74)  62 (39)  24 (15)  47 (30)  63 (40)  40 (25)  88 (55)  56 (35)  107 (67)  43 (27)  49 (31)  23 (14)  27 (17)  4 (3-6)  111 (70) | 8 (73)  2 (18)  1 (9)  3 (27)  4 (36)  3 (27)  6 (55)  5 (45)  6 (55)  3 (27)  4 (36)  2 (18)  2 (18)  4 (3-6)  7 (64) |
| Elevated CRP | 102/128 (80) | 10/10 (100) |
| Clonal hematopoiesis | 61/108 (56) | 5/7 (71) |
| Mixed histiocytosis  ECD-LCH  ECD-RDD | 33 (21)  22 (14)  12 (8) | 1 (9)  1 (9)  0 (0) |
| Second cancer  Hematologic  Solid | 51 (32)  35 (22)  24 (15) | 3 (27)  2 (18)  1 (9) |
| Somatic mutations  *BRAF*^V600E^  *MAP2K1*  *NRAS*  *KRAS*  No mutation | 53/153 (35)  43/153 (28)  7/153 (5)  6/153 (4)  46/153 (30) | 4 (36)  3 (27)  0 (0)  0 (0)  4 (36) |

Data are n (%) or median (IQR)

None of these above-reported features was statistically different between the two groups

*Abbreviations: CNS, central nervous system; CRP, C-reactive protein; ECD, Erdheim-Chester disease; LCH, Langerhans cell histiocytosis; RDD, Rosai-Dorfman-Destombes disease*

**Table S2.** Characteristics of patients who progressed on MEKi treatment

| **Sex, age at diagnosis (years)** | **Somatic mutation** | **Organ involvement** | **Previous lines of treatment** | **Time on MEKi (months)** |
| --- | --- | --- | --- | --- |
| F, 63 | *BRAF*^V600E^ | Long bones, CNS, large vessels, perirenal, skin | BRAFi | 5 |
| F, 24 | None | Long bones, facial/orbit, large vessels | anti-IL1 | 2 |
| M, 37 | *BRAF*^V600E^ | Long bones, CNS, facial/orbit, heart, perirenal | IFNa, BRAFi | 33 |
| M, 56 | *BRAF*^V600E^ | Long bones, CNS, perirenal | BRAFi | 6 |
| M, 73 | None | Long bones, lungs, perirenal, skin | IFNa | 19 |
| M, 67 | None | Facial/orbit, large vessels, perirenal, skin | IFNa | 42 |
| M, 45 | *MAP2K1*  (class 3) | Long bones, CNS, lungs, perirenal, skin, pituitary | Chemotherapy | 68 |
| M, 65 | *MAP2K1* | CNS, heart, large vessels | None | 4 |
| M, 61 | None | Large vessels, perirenal | None | 30 |
| M, 56 | *BRAF*^V600E^ | Long bones, CNS, large vessels, perirenal, skin, pituitary | IFNa, BRAFi | 62 |
| M, 68 | *MAP2K1* | Long bones, heart, lungs, perirenal | BRAFi+MEKi | 1 |
| M, 53 | None | Long bones, CNS, perirenal | None | 37 |

*Abbreviations: CNS, central nervous system; BRAFi, BRAF inhibitors; IFNa, interferon-alpha; MEKi, MEK inhibitors*

**Table S3.** Predictors of time-dependent response to MEKi in the 159 evaluable patients

|  | Univariable | | Multivariable | |
| --- | --- | --- | --- | --- |
|  | HR (95% CI) | *p*-value | HR (95% CI) | *p*-value |
| Age at diagnosis | 0.999 (0.986-1.013) | 0.887 |  |  |
| Female sex | 1.001 (0.665-1.504) | 0.998 |  |  |
| Cardiovascular comorbidities  Smoking  T2DM  Obesity  Dyslipidemia  Hypertension  CAD | 1.396 (0.669-2.914)  1.280 (0.803-2.041)  0.761 (0.453-1.315)  0.734 (0.475-1.132)  0.789 (0.539-1.156)  0.892 (0.565-1.407) | 0.374  0.299  0.340  0.162  0.224  0.622 | 0.441 (0.238-0.819) | 0.009 |
| Organ involvement  Long bone  CNS  Neurodegeneration  Facial/orbit  Heart  Right atrium  Large vessel  Lung  Perirenal  Hypothalamic/Pituitary  Skin  Gastrointestinal  Lymph nodes  Number of involved sites  ≥4 involved sites | 0.765 (0.504-1.159)  0.605 (0.409-0.897)  0.524 (0.286-0.957)  1.075 (0.721-1.604)  1.319 (0.900-1.933)  1.643 (1.073-2.517)  1.388 (0.948-2.031)  0.915 (0.615-1.361)  0.776 (0.526-1.146)  0.907 (0.599-1.373)  1.139 (0.773-1.678)  1.739 (1.071-2.826)  1.331 (0.827-2.141)  1.031 (0.931-1.141)  1.091 (0.720-1.656) | 0.206  0.012  0.036  0.723  0.156  0.022  0.091  0.661  0.203  0.645  0.510  0.025  0.239  0.562  0.681 | 3.959 (1.956-8.014)  2.466 (1.279-4.753) | 0.001  0.007 |
| Elevated CRP | 0.839 (0.497-1.418) | 0.513 |  |  |
| Clonal hematopoiesis | 0.988 (0.626-1.560) | 0.960 |  |  |
| Mixed histiocytosis  ECD-LCH  ECD-RDD | 0.922 (0.526-1.616)  0.846 (0.412-1.739) | 0.778  0.649 |  |  |
| Second cancer  Hematologic  Solid | 0.894 (0.564-1.415)  1.321 (0.806-2.165) | 0.632  0.269 |  |  |
| Somatic mutations  *BRAF*^V600E^  *MAP2K1*  Other  Any mutation | 0.741 (0.493-1.113)  1.227 (0.814-1.849)  1.093 (0.570-2.098)  0.912 (0.615-1.353) | 0.149  0.329  0.788  0.646 |  |  |
| Treatment line | 0.854 (0.666-1.095) | 0.213 | 0.545 (0.358-0.830) | 0.005 |

*Abbreviations: T2DM. type-2 diabetes mellitus; CAD. coronary artery disease; CNS. central nervous system; CRP. C-reactive protein; ECD. Erdheim-Chester disease; LCH. Langerhans cell histiocytosis; RDD. Rosai-Dorfman-Destombes disease*
